# Comparing the Mini-BESTest with the Berg Balance Scale to Evaluate Balance Disorders in Parkinson's Disease

**DOI:** 10.1155/2012/375419

**Published:** 2011-10-24

**Authors:** Laurie A. King, Kelsey C. Priest, Arash Salarian, Don Pierce, Fay B. Horak

**Affiliations:** ^1^Department of Neurology, Oregon Health & Science University, Portland, OR 97239, USA; ^2^Division of Biostatistics, Department of Public Health and Preventive Medicine, Oregon Health & Science University, Portland, OR 97239, USA

## Abstract

*Objective*. The purpose of this study was to explore the usefulness of the Mini-BESTest compared to the Berg Balance Scale in evaluating balance in people with PD of varying severity. We evaluated (1) the distribution of patients scores to look for ceiling effects, (2) concurrent validity with severity of disease, and (3) the sensitivity/specificity of separating people with or without postural response deficits. *Subjects*. Ninety-seven people with PD were tested for balance deficits using the Berg, Mini-BESTest, Unified Parkinson's Disease Rating Scale (UPDRS) III and the Hoehn & Yahr (H&Y) disease severity classification. *Setting*. Clinical research facility at Oregon Health & Science University. *Results*. The Mini-BESTest is highly correlated with the Berg (*r* = 0.79, *P* < 0.001), but avoids the ceiling compression effect of the Berg for mild PD (skewness −2.30 Berg, −0.93 Mini-BESTest). Consequently, the Mini-BESTest is more effective than the Berg for predicting UPDRS Motor score (*P* < 0.001 Mini-BESTest versus *P* = 0.86 Berg), and for discriminating between those with and without postural response deficits as measured by the H&Y (ROC differential *P* = 0.06). *Conclusion*. The Mini-BESTest is a promising tool for discerning balance deficits in patients with PD, most importantly those with more subtle deficits.

## 1. Introduction

Postural instability and balance deficits are one of the most debilitating impairments associated with chronic neurological disease, such as Parkinson's disease (PD) [1]. The most commonly used clinical test of balance severity in people with PD is the Berg Balance Scale (Berg) [2]. The Berg, originally designed for use in the frail elderly, is a 14-item test that focuses on a variety of self-initiated tasks related to everyday function such as sit-to-stand and functional reach forward. The Berg has excellent reliability and is somewhat correlated with severity of PD, as measured with the Unified Parkinson Rating Scale (UPDRS) [3, 4]. However, the Berg has limitations such as documented ceiling effects [5–7] and problems with underutilization and redundancy of categories due to the rating scale [8, 9]. These particular limitations are important considerations when evaluating patients with mild neurological deficits, who are easy to underidentify and therefore less likely to receive rehabilitation. 

Such documented limitations of the Berg have led many clinicians to do more than one validated balance assessment in order to identify deficits that may respond to treatment. Recently, a new and more comprehensive clinical balance test, the Balance Evaluation Systems Test (BESTest), has been developed that is essentially a battery of balance and mobility tests, borrowed from other validated tests such as the Berg and Dynamic Gait Index. The BESTest was uniquely designed as a comprehensive clinical tool for evaluating six different balance control systems: biomechanical, stability limits/verticality, anticipatory, reactive, sensory orientation, and stability in gait. Such system-specific assessment is helpful in directing treatment and to ensure that a meaningful deficit is not overlooked. The BESTest has good interrater reliability [10] and good validity in discerning fallers from nonfallers in patients with PD [11]. 

The BESTest, though comprehensive, valid, and reliable, is lengthy to administer and may not always be practical in a busy clinical setting. Thus, a shorter version of the BESTest, the Mini-BESTest, was developed using psychometric techniques to reduce item redundancy and simplify scoring [12]. This shorter version has excellent interrater (ICC ≥ 0.91), and test-retest (ICC ≥ 0.88) reliability and similar in length to the Berg [13]. However it is currently unknown how the Mini-BESTest compares with the Berg in detecting balance deficits in the PD population. 

The purpose of this study was to explore the usefulness of the Mini-BESTest compared to the Berg in evaluating balance in people with PD of varying severity. Specifically, we evaluated (1) the distribution of patients scores to look for ceiling effects, (2) concurrent validity with severity of disease, and (3) the sensitivity/specificity of separating people who do or do not have postural response deficits. 

## 2. Methods

Ninety-seven participants with idiopathic PD participated in the study. These participants were part of either a larger clinical study examining prospective fall risk or an exercise efficacy study. Therefore, the group here represents a convenience sample of participants with PD, and the data for this paper was taken from their baseline visits. Inclusion criteria: all people in the study were diagnosed with idiopathic PD by a movement disorders neurologist. People were excluded from the study if they presented with cognitive impairment, prior orthopedic injuries, or impairments that could interfere with mobility such as artificial joints or peripheral neuropathy or prior brain surgery such as a pallidotomy or deep brain stimulation. All participants signed informed consent forms approved by the Oregon Health & Science University Institutional Review Board. All work was conducted in accordance with the declaration of Helsinki (1964). 

All participants came in for an assessment of their balance and mobility which included both clinical and instrumented testing. The data presented in this paper is taken from the clinical scales: the Unified Parkinson's Disease Rating Scale (UPDRS) III Motor section, Hoehn & Yahr (H&Y) disease severity classification, and the Berg and the Mini-BESTest. The testing was performed in the same order for each participant, and rest breaks were given as needed to avoid fatigue. Other balance and gait assessments conducted during testing that were not included in this analysis included gait and sway analysis using wearable inertial sensors. Testing was conducted at the Oregon Clinical Translational Research Institute at Oregon Health & Science University. All participants took their PD medication as normally indicated and were tested in the ON state. All of the participants except for two were currently taking some form of PD medication. The testing was administered by a trained examiner, overseen by a physical therapist. Participant characteristics are outlined in Table 1.

### 2.1. Clinical Tests

#### 2.1.1. Mini-BESTest

The Mini-BESTest test is a 14-item test that focuses on dynamic balance, specifically anticipatory transitions, postural responses, sensory orientation, and dynamic gait [12]. Each item is scored from (0–2); a score of 0 indicates that a person is unable to perform the task while a score of 2 is normal. The best score is the maximum amount of points, being 28.

#### 2.1.2. Berg Balance Scale (Berg) [2]

The Berg is a 14-item test designed to measure the balance of older adults by assessing their performance of specific functional tasks [14]. Each task is scored from (0–4), for a maximum of 56 points. The test indicates that a score of 41–56 is associated with a low fall risk, 21–40 with a medium fall risk, and 0–20 with a high fall risk [14].

#### 2.1.3. Unified Parkinson's Disease Rating Scale (UPDRS)

Disease severity was evaluated using the UPDRS III motor component [15]. This test has a maximum score of 108; each item is scored from 0-not affected through 4-most severely affected. 

#### 2.1.4. Hoehn and Yahr (H&Y)

Postural response deficits were identified as patients scoring 3 to 4 in the H&Y scale. [16]. A score of 3 and above indicates postural instability as defined by an abnormal stepping response to a backwards pull on the shoulders. The H&Y scale is the most commonly used method for evaluating the severity of PD [17], and the scale ranges from 0 (no symptoms of PD) to 5 (wheelchair bound).

### 2.2. Statistics

The STATA statistical package was used for both calculations and graphics [18]. We describe the Berg and Mini-BESTest data for the 97 participants, using histograms and a scatter plot displaying the association between the two variables. We used the bootstrap method to assess a *P* value for the skewness [19]. We also carried out a regression of UPDRS jointly on the two scores for the Berg and Mini-BESTest. This regression provided information on the relative contributions of the Berg and Mini-BESTest for predicting the UPDRS, each adjusted for the other measure using added variable or partial correlation plots that show the extent of information in each test that is not conveyed by the other test [20]. Finally, we considered the relative performance of the Berg and Mini-BESTest in terms of receiver operating characteristic (ROC) curves for classifying people into two groups based on a threshold for the H&Y score, to discriminate between mild PD (H&Y 1-2) versus more severe PD (H&Y 3-4) [21]. 

## 3. Results

### 3.1. Distribution of Scores and Relation between Berg and Mini-BESTest

**Figure 1:**
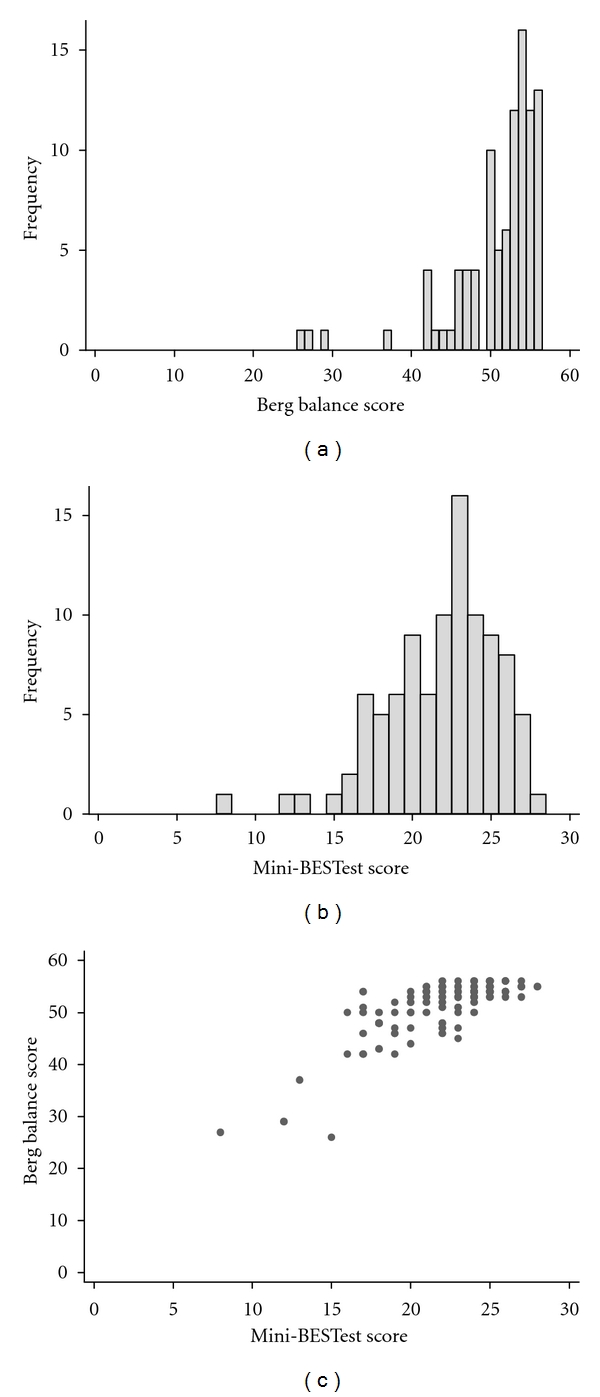
Distribution of scores for the Berg Balance scale (a) and Mini-BESTest test (b), along with a scatter plot showing their relationship to one another (c) for 97 patients with Parkinson's disease.

The distribution of scores among the 97 participants with PD on the Mini-BESTest differed significantly from the Berg (Figure 1). The Mini-BESTest scores were significantly less skewed than the Berg (Berg skewness = −2.3 versus Mini-BESTest skewness = 0.93; *P* < 0.001). Using the bootstrap method, we found that, sampling from a population with the shape of the Mini-BESTest histogram, the chance would be less than 0.001 of obtaining a skewness as extreme as that seen for Berg. The scatter plot in Figure 1(c) shows the relationship between the two measures. 

The Mini-BESTest and Berg correlate significantly (*r* = 0.79; *P* < 0.001). However, people scoring the highest values in the Berg (i.e., 52–56; those with scores in the clinically accepted range as “normal”) had scores representing approximately half of its maximum range in the Mini-BESTest. This suggests that the Mini-BESTest “spreads out” the compression (ceiling effect) at the top end of the Berg. 

### 3.2. Relationship to PD Severity

**Figure 2:**
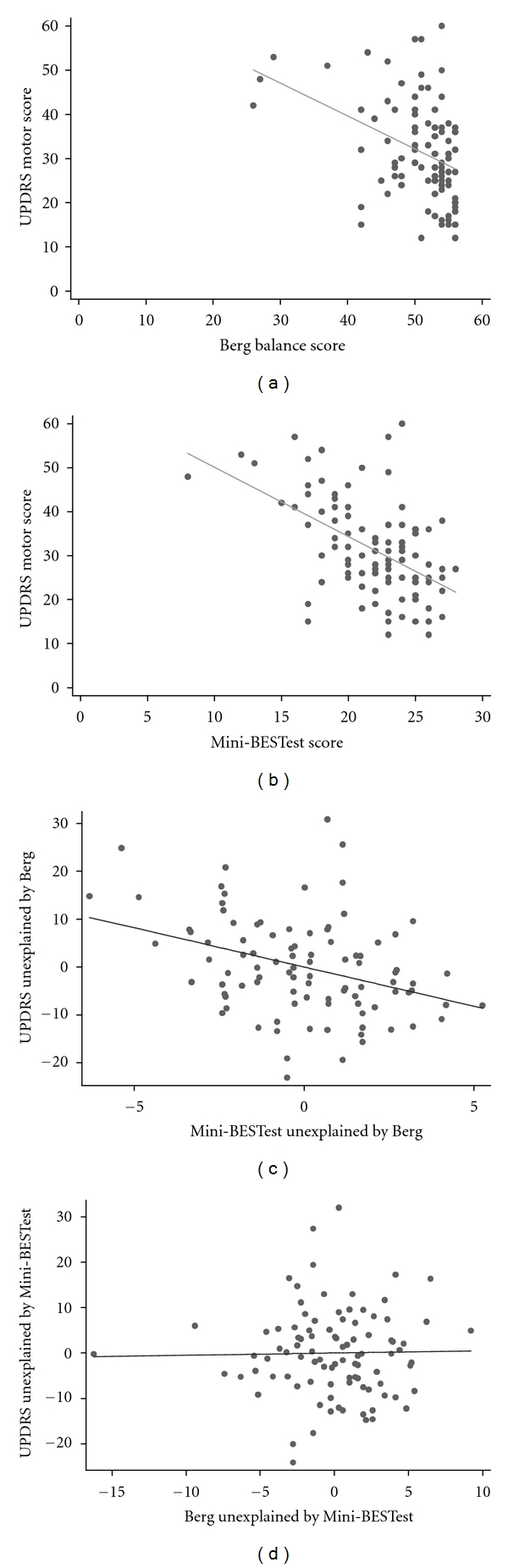
Scatter plots showing the relationship of the UPDRS motor score to (a) Berg Balance scale, and (b) Mini-BESTest score. Lower panels show (c) added value of the Mini-BESTest over Berg and, (d) added value of Berg over Mini-BESTest for predicting UPDRS motor score.

Both the Mini-BESTest and Berg were moderately correlated with disease severity as measured by the UPDRS. Figures 2(a) and 2(b) display the individual regression lines, indicating that the Berg and the Mini-BESTest each have a significant correlation to the UPDRS (−0.39 and −0.51, *P* > 0.001, respectively). 

Using a multiple regression of the UPDRS on *both* the Mini-BESTest and the Berg, we determined how much either test compliments the other in the prediction of disease severity. For linear regression prediction of the UPRDS, the Berg did not provide statistically significant information in addition to the Mini-BESTest (*t* = 0.18; *P* = 0.86). In contrast, the Mini-BESTest provided significant information in addition to the Berg (*t* = −3.7; *P* = 0.001) to predict severity of disease. The added variable plot in Figure 2(c) shows the extent of information in the Mini-BESTest for predicting UPDRS, beyond that provided by the Berg. This was significant (*P* < 0.001). The added variable plot in Figure 2(d) shows the extent of information in the Berg for predicting UPDRS, beyond that provided by Mini-BESTest. This was not statistically significant (*P* = 0.86).

### 3.3. Identifying Mild Deficits

**Figure 3:**
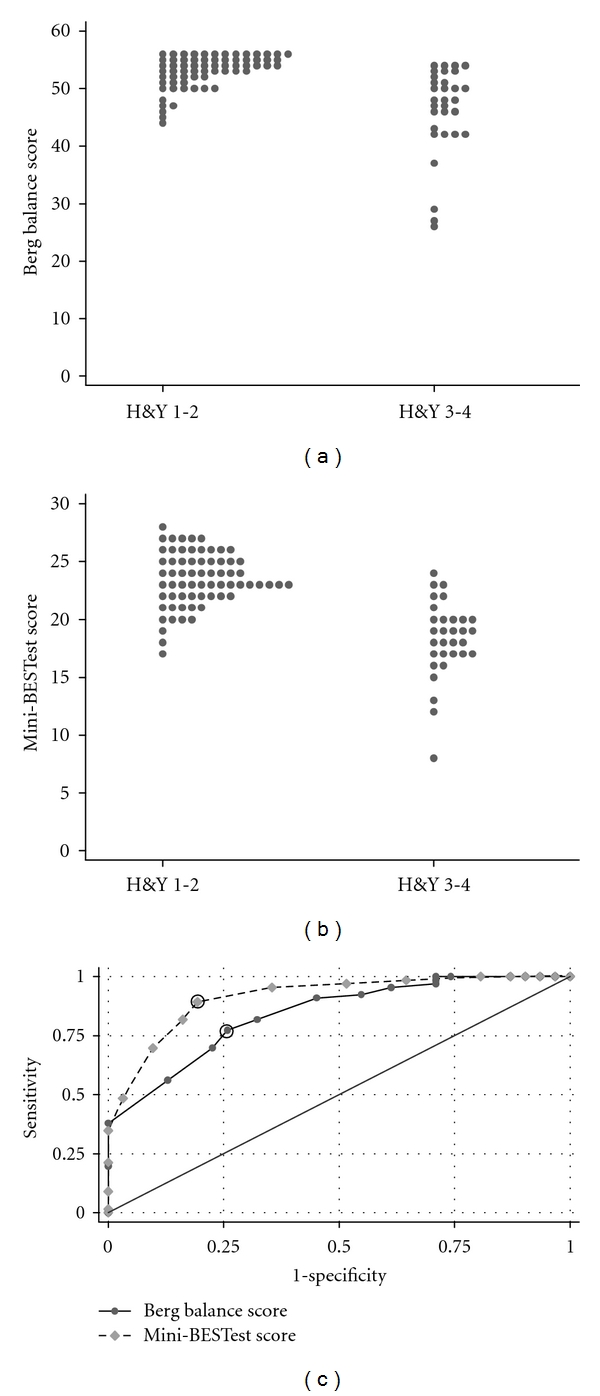
(a) and (b) show the distribution of scores on the Berg Balance Scale (a) and the Mini-BESTest (b) when patients were separated into those without postural response deficits (H&Y 1-2) and those with postural deficits (H&Y 3-4). (c) compares the receiver operator characteristics of the Berg Balance Scale and the Mini-BESTest to differentiate people with and without postural response deficits as measures by H&Y classification.

We compared the ability of the Berg and Mini-BESTest to differentiate PD patients with and without clinical balance deficits. Participants with and without clinical balance deficits were classified using H&Y: H&Y 1-2 and H&Y 3-4. A score of H&Y 3 and 4 identifies people with abnormal postural stepping response to the backwards pull test or observable postural instability. Though the mean H&Y score was 2.3, the range was 1–4. Roughly one third (31 of 97) of the participants had a H&Y of 3 or above, indicating postural instability as defined by H&Y. Figures 3(a) and 3(b) compare the distributions of Berg and Mini-BESTest scores for people with H&Y 1-2 versus H&Y 3-4. ROC analysis was done to test the discriminative ability of these different balance tests to differentiate those people with and without abnormal postural responses. 

The area under the ROC curves (AUC) differed for the tests; the AUC for the Berg = 0.84 ± 0.04 and the AUC for the Mini-BESTest = 0.91 ± 0.03. The 2-sided *P*-value for testing equality of the two AUC values was 0.05. A suggested cut-off point for the Mini-BESTest to differentiate those with and without postural response deficits is > 21, yielding (sensitivity, specificity) = (89%, 81%). The nearest point to this for the Berg is ≥52, yielding (Sensitivity, Specificity) = (77%, 74%). The points corresponding to these cut-off points are indicated by circles in Figure 3(c). 

### 3.4. Most Difficult Items for People with PD

Individual items from both the Berg and the Mini-BESTest were ranked in order of difficulty for the whole population of people with PD within this study and classified as “difficult” if a person had a score less than perfect on that item (2 = perfect; 1 = some difficulty, or 0 = cannot perform) (Table 2). We found that 72% (10 out of 14) items on the Mini-BESTest presented some difficulty to at least one-third of the group versus only 36% (5 out of 14 items) in the Berg.

## 4. Discussion

The results from this study suggest that the Mini-BESTest may be more useful than the Berg in evaluating balance disorders in patients with PD, especially in those with mild PD or more subtle balance deficits. Specifically, results showed that (1) although the Mini-BESTest had a high correlation with the Berg, it did not have the same ceiling effects; (2) both the Berg and Mini-BESTest correlated with PD severity but the Mini-BESTest added value to the Berg score; (3) the Mini-BESTest test had better sensitivity/specificity then the Berg to identify people with abnormal postural responses. 

The high correlation of the Mini-BESTest with the Berg supports concurrent validity since the Berg remains one of the most commonly used clinical scales for balance assessment in people with PD. But importantly, we found very different test score distributions across patients with varied levels of severity. Though neither test had a normal distribution, the Mini-BESTest was significantly less skewed, indicating that there are less ceiling effects as has been shown previously with the Berg [22]. These results are not surprising since the Berg was originally intended for frail elderly and remains an excellent measure of balance deficits for those with more severe PD. 

The high sensitivity of the Mini-BEST is important for clinicians who see patients with mild balance deficits who are seeking to identify and treat potentially preventable mobility problems early in the disease progression. 

The Berg has been shown to have excellent test-retest reliability [3] and to correlate significantly with disease severity in PD [23], and our results support the relationship with the UPDRS. Both exercise and physical therapy have been shown to improve UPDRS scores. Therapists need measures that reflect improvements with intervention so comparing the Mini-BESTest with the UPDRS establishes concurrent validity of the new test with an established one. The novel information obtained from our study is that while both the Berg and Mini-BESTest correlate with disease severity, the Mini-BESTest adds value not included in the Berg, but the Berg does not add value to the Mini-BESTest. These findings suggest that the Mini-BESTest distinguishes among PD subjects who all get similar, high scores in the Berg, and this information can add to the prediction of disease severity. A previous study demonstrated the Berg to be useful in identifying balance impairments in people with very severe PD (i.e., H&Y 4), but it could not discriminate subgroups of H&Y scores successfully [24]. Here, we found similar results in that the Mini-BESTest was more successful than the Berg at discriminating subgroups of PD severity as measured by the H&Y scale. Franchignoni et al. examined the clinimetric properties of the Berg with 57 participants with PD [9]. They found excellent internal consistency, good correlations to other scales of disease severity, and quality of life, all agreeing with previously published work [4]. However, they did find, using a Rasch analysis, that some rating categories were not used and others were underutilized. The authors suggested that improving the rating scale structure would improve the test. The same type of Rasch analysis was performed on the full BESTest to obtain the shortened Mini-BESTest that excludes redundant or underused items [12]. 

The cut-off point of the Mini-BESTest for identifying patients with PD who had problems with the “Pull test” (i.e., H&Y score of at least 3) was a score of 21. It is interesting that a similar cut-off point for the Mini-BESTest for identifying patients with PD who fall was a score of 20 [13]. Both the Mini-BESTest and the Berg were sensitive (89% and 77%, respectively) and specific (81% and 74%, respectively) in differentiating those with and without postural response deficits. Similarly, the Mini-BESTest was also shown to be sensitive (88%) and specific (78%) in identifying PD patients with a history of falls [13]. 

It has been suggested that postural instability in PD is multifactorial, therefore, a multitude of tests should be administered by physical therapists [25, 26]. For example, the Berg does not include tests of postural reactions or dynamic gait, and, therefore, some deficits may be missed. Since the Mini-BESTest is essentially a combination of tests, this may be a reason it successfully identified people with mild balance deficits. As outlined in Table 2, each test item primarily tests one of 4 categories of balance: anticipatory, dynamic gait, reactive control, and sensory orientation. The Berg was not designed with such systems in mind but if a system categorization is assigned to each item, the Berg items primarily evaluate anticipatory and sensory contributions to balance. There are two additional systems that the Mini-BESTest evaluates, dynamic gait, and reactive postural control, this may explain the added variable plot being significant for the Mini-BESTest adding value to the Berg in relating to disease severity. In other words, the Mini-BESTest usefully distinguishes among those persons that are overly range compressed in the Berg. If a clinician is using the Berg for their PD patients, it may be beneficial to augment testing with the Dynamic Gait Index and the Pull test from the UPDRS. Dynamic gait (cognitive task with gait) and reactive postural control (response to perturbation) items were the most difficult items for people with PD, balance systems that are not assessed using the Berg. 

Clinicians commonly use single-limb stance for balance assessment. An example of a difference between testing items in the Berg and Mini-BESTest is the assessment of the single-limb stance (item #14 Berg, item #3 Mini-BESTest). In the Berg, the participant chooses either leg, and it is only this side that is assessed. Comparatively, the Mini-BESTest assesses both the left and right leg and records the worst side. In this study, when the Berg was used, assessing only one leg, 39% of the participants had some observable difficulty. When the Mini-BESTest was used, assessing both left and right leg, 81% of the participants had some difficulty. Therefore, clinicians should test standing balance on both sides.

This study was limited to people with PD so it needs to be repeated in patients with other pathologies affecting balance control. One potential limitation is that the order of testing was not randomized so fatigue may have factored into test performance. However, participants were given frequent rest breaks to avoid fatigue. 

In conclusion, the Mini-BESTest is a novel, useful, and easy to administer tool for balance assessment. Although the Mini-BESTest had a high correlation with the Berg, it did not have the same ceiling effects. Furthermore, both the Berg and Mini-BESTest correlated with PD severity but the Mini-BESTest added value to the Berg score in predicting disease severity. Finally, the Mini-BESTest test had better sensitivity/specificity than the Berg to identify people with abnormal postural responses. Taken together, these findings suggest that the Mini-BESTest is a promising tool for discerning balance deficits in patients with mild to severe PD.

## Figures and Tables

**Table 1:** Participant characteristics.

Variables	Mean	SD	Range
UPDRS III	31.6	11.2	12–60
Hoehn & Yahr	2.3	0.6	1–4
Age (yr)	65.6	7.1	47–83
Time since dx (yr)	6.5	5.0	0–23
Height (cm)	172.6	9.5	152–198
Weight (kg)	79.2	15.6	43–120
Gender	Male 59		Female 38

**Table 2:** The Berg and Mini-BESTest individual items ranked from most difficult to least based on the % of participants with PD who did not have normal scores. Difficulty with the test was determined if the participant did not receive a perfect score.

Berg test item	Percentage (% with difficulty)	Mini-BESTest item	Percentage (% with difficulty)	System (Mini-BEST)
Turning to look behind	70.1	Rise to toes	86.6	Anticipatory
Standing with one foot in front	42.3	Single leg	81.4	Anticipatory
Reaching forward with outstretched arms	40.2	TUG w/Cog	54.6	Gait
Standing on one foot	39.2	Pivot turn	51.5	Gait
Turn 360 degrees	30.9	Eyes Closed/foam	46.4	Sensory
Placing alternate foot on stool	27.8	Obstacle during Gait	46.4	Gait
Standing to sitting	11.3	Turn head with gait	41.2	Gait
Retrieving object from the floor	9.3	Incline eyes closed	33	Sensory
Sitting to standing	5.2	Backwards recovery	29.9	Postural
Standing with feet together	4.1	Lateral recovery	29.9	Postural
Transfers	4.1	Change pace gait	13.4	Gait
Standing with eyes closed	3.1	Forward recovery	13.4	Postural
Standing unsupported	3.1	Sit to stand	6.2	Anticipatory
Sitting unsupported	0	Eyes open stance	2.1	Sensory
